# Evaluation of peripheral basophil activation during exercise provocation test for desensitized patients

**DOI:** 10.3389/falgy.2023.1298137

**Published:** 2023-12-22

**Authors:** Jun Kunizaki, Shiro Sugiura, Akira Sakai, Miyuki Teshigawara, Atsushi Makino, Yoshihiro Takasato, Teruaki Matsui, Yasuto Kondo, Komei Ito

**Affiliations:** ^1^ Department of Allergy, Allergy and Immunology Center, Aichi Children’s Health and Medical Center, Obu, Japan; ^2^ Department of Pediatrics, NTT East Sapporo Hospital, Sapporo, Japan; ^3^ Department of Pediatrics, Hamamatsu Medical Center, Hamamatsu, Japan; ^4^ Department of Pediatrics, Bantane Hospital, Fujita Health University, Aichi, Japan

**Keywords:** oral immunotherapy, exercise-induced allergic reactions on desensitization, exercise provocation test, basophil activation, CD203c, CD63

## Abstract

Some food allergic patients who have undergone oral immunotherapy develop exercise-induced allergic reactions on desensitization (EIARDs). This study investigated basophil activation status during the exercise provocation test (EPT) performed to diagnose EIARD. EPT was performed on 20 participants, and *in vivo* basophil activation status was analyzed using activation markers CD203c and CD63. The results showed that there was no significant difference between EPT-positive and negative subjects for basophil activation status throughout EPT. Consequently, *in vivo* basophil activation after ingestion of the causative food may not be associated with EIARDs. New tests are desired for predicting EIARDs.

## Introduction

Although oral immunotherapy (OIT) is an effective treatment for inducing desensitization in patients with persistent food allergies (1), they sometimes develop allergic reactions induced by exercise after food ingestion. This occurs even after desensitization, and is referred to as exercise-induced allergic reactions on desensitization (EIARDs) (2). The pathophysiology of EIARDs remains unclear. Dua et al. reported that exercise reduced the symptom-provoked threshold by 45% in patients allergic to peanuts (3). Therefore, even patients desensitized to a certain amount of the causative antigen after OIT could develop allergic symptoms through lowering of the symptom-provoked threshold with exercise. Because of the augmented small intestinal permeability with exercise, increasing the amount of the absorbed causative antigen might trigger symptoms (4).

Another typical exercise-induced allergic symptom is food-dependent exercise-induced anaphylaxis (FDEIA). Exercise may enhance the clinical reactions by both lowering the threshold and increasing the severity of the allergic reaction (5, 6). These findings indicate that the pathophysiology of EIARDs is at least partially similar to that of FDEIA.

The exercise provocation test (EPT) for diagnosis of EIARDs is important for determining if patients can ingest causative food safely in daily life, given that exercise after meals is generally inevitable. The test itself is burdensome and may cause severe allergic reactions. Therefore, alternative tests are needed.

Basophils play an important role in IgE-mediated allergic reactions. Recent reports showed that basophil activation is observed after the ingestion of causative foods, even before the occurrence of allergic reactions (7, 8). Based on this finding, we hypothesized that basophils are activated by ingestion of the causative food before the onset of symptoms of EIARDs and that exercise induced further basophil activation leading to EIARDs. If the hypothesis is proven correct, evaluation of the activation status of basophils might help the diagnosis of EIARDs even without EPT. Therefore, we analyzed the basophil-activation status during EPTs for diagnosing EIARDs.

## Method

Twenty patients underwent EPTs on their desensitization status between April 2018 and January 2019 after rush (2) or slow (9) oral immunotherapy for each causative food. We judged desensitization to the causative foods by daily ingesting full dose (10) of causative antigen or more without symptoms at least 2 months before EPT. Two patients were allergic to hen's egg, 11 to cow's milk, 5 to wheat, 1 to peanuts, and 1 to sesame seeds. We measured total and specific IgE titers for each causative antigen within 3 months before the EPT. The patient's characteristics are shown in Table 1.

**Table 1:** Patient profile and results of the exercise provocation test.

No.	Age (year)	Sex	OIT	Allergen	Total IgE (IU/ml)	Crude IgE (*UA*/ml)	Component IgE (*UA*/ml)	Dose	Result	Total score	Symptoms	Treatment
1	15	M	Slow	Hen's egg	6718	33.2	3.66	40 g boiled egg white	Positive	5	Hives	–
2	10	M	Rush	Hen's egg	699	9.66	2.56	40 g boiled egg white		5	Hives	–
3	14	M	Rush	Cow's milk	145	0.77	0.82	200 ml Cow's milk		30	Hives, intermitting coughing, sleep	H, S
4	12	M	Rush	Cow's milk	1084	8.76	13.1	200 ml Cow's milk		10	Hives	–
5	8	M	Slow	Cow's milk	2276	7.74	2.65	200 ml Cow's milk		10	Mild wheezing	I
6	15	M	Rush	Cow's milk	554	1.63	1.05	200 ml Cow's milk		10	Hives	–
7	13	M	Rush	Wheat	282	35.4	3.83	50 g plain bread		40	Hives, apparent wheezing	H, S, I
8	14	M	Slow	Wheat	921	3.22	0.50	200 g udon noodle		10	Mild wheezing	I
9	10	F	Rush	Wheat	247	8.10	0.34	200 g udon noodle		5	Hives	–
10	9	F	Rush	Cow's milk	7588	26.3	29.9	200 ml Cow's milk	Negative	0	Nothing	Nothing
11	14	M	Rush	Cow's milk	6718	8.62	5.27	400 ml Cow's milk				
12	14	M	Rush	Cow's milk	1216	5.97	7.99	200 ml Cow's milk				
13	10	M	Rush	Cow's milk	372	3.58	1.72	200 ml Cow's milk				
14	10	M	Rush	Cow's milk	615	1.32	1.32	200 ml Cow's milk				
15	11	F	Rush	Cow's milk	709	0.76	1.20	200 ml Cow's milk				
16	8	M	Slow	Cow's milk	82	0.47	0.59	350 ml Cow's milk				
17	9	M	Rush	Wheat	927	28.0	1.62	200 g udon noodle				
18	12	M	Rush	Wheat	651	23.6	0.62	200 g udon noodle				
19	12	M	Rush	Peanut	1521	10.1	3.40	3 g peanut				
20	14	M	Rush	Sesame	994	5.69	–	4 g sesame seeds				

OIT, oral immunotherapy; M, male, F, female; H, anti-histamine; S, steroid; I, Inhaling a beta-stimulator or adrenaline; Crude IgE, specific IgE titer(ImmunoCAP) to Egg white, Milk, Wheat, Peanut and Sesame; Component IgE, specific IgE titer(ImmunoCAP) to Ovomucoid, Casein, Omega-5-gliadin, and Ara h 2.

**Figure 1:**
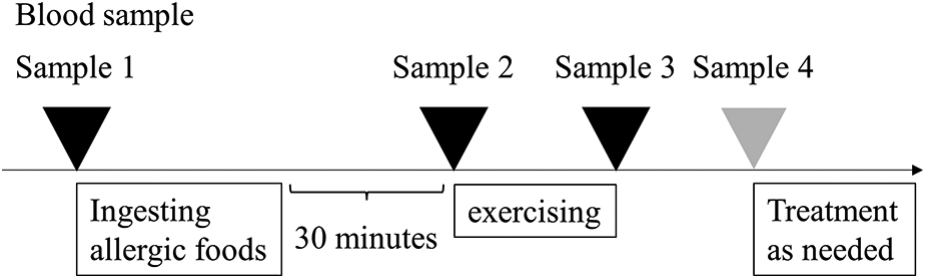
Schematic illustration of the exercise provocation test. The exercise provocation test was performed 30 min after consuming allergic foods. Sample 1; before eating, Sample 2; 30 min later, Sample 3; immediately after exercise, Sample 4; at the peak of the provoked symptoms.

EPTs were performed in our hospital, as previously reported (2). Initially, patients consumed full doses (10) of the causative foods, and after 30 min engaged in free running for approximately 10 min to achieve a target heart rate of >180 beats/min. The provoked symptoms were scored using the total score (TS) of “Anaphylaxis Scoring Aichi” (2, 9). Blood samples were collected during the EPTs: before eating (sample 1), 30 min later (sample 2), immediately after exercise (sample 3), and at the peak of the provoked symptoms (sample 4) (Figure 1). To assess the basophil-activation status after causative food ingestion, peripheral blood samples were incubated with phosphate-buffered saline without further ex vivo stimulation. The *in vivo* basophil-activation status was evaluated using the Allergenicity kit (Beckman Coulter, Brea, CA) with the anti-CD63 antibody (Anti-Hu CD63-APC; EXBIO, Praha, Vestec, Czech Republic), following the protocol defined by the kit (Supplementary Figure S1). We compared the changes in the activation status by both mean fluorescence intensity (MFI) and CD203c high or CD63 high basophils, which were gated over the top 5% of the expression in comparison to sample 1. In addition, we measured plasma histamine, serum tryptase levels (LSI Medicine Corporation, Tokyo, Japan), and basophil count, which were evaluated using an automated analyzer.

The primary outcome was the change in the expressions of CD203c, CD63, and their MFI on no-stimulation basophils from sample 1 to sample 2 in the patients with a positive/negative result of the EPTs. Secondary outcomes included the change in the expressions of CD203c, CD63, and their MFI of all samples, plasma histamine, tryptase, and peripheral blood basophil counts. To evaluate the validity of the measurement system, we assessed the *in vitro* basophil-activation status in sample 1 under stimulation by anti-IgE antibodies or each causative antigen. We used the EZR software program (11) (Saitama Medical Center, Jichi Medical University, Saitama, Japan) to analyze data and determined statistical significance (*P* < 0.05) using the Mann–Whitney U or Wilcoxon rank sum tests. This study was approved by the Institutional Review Board of Aichi Children's Health and Medical Center (20170791). Written informed consent was obtained from all patients before enrollment.

## Results

**Figure 2:**
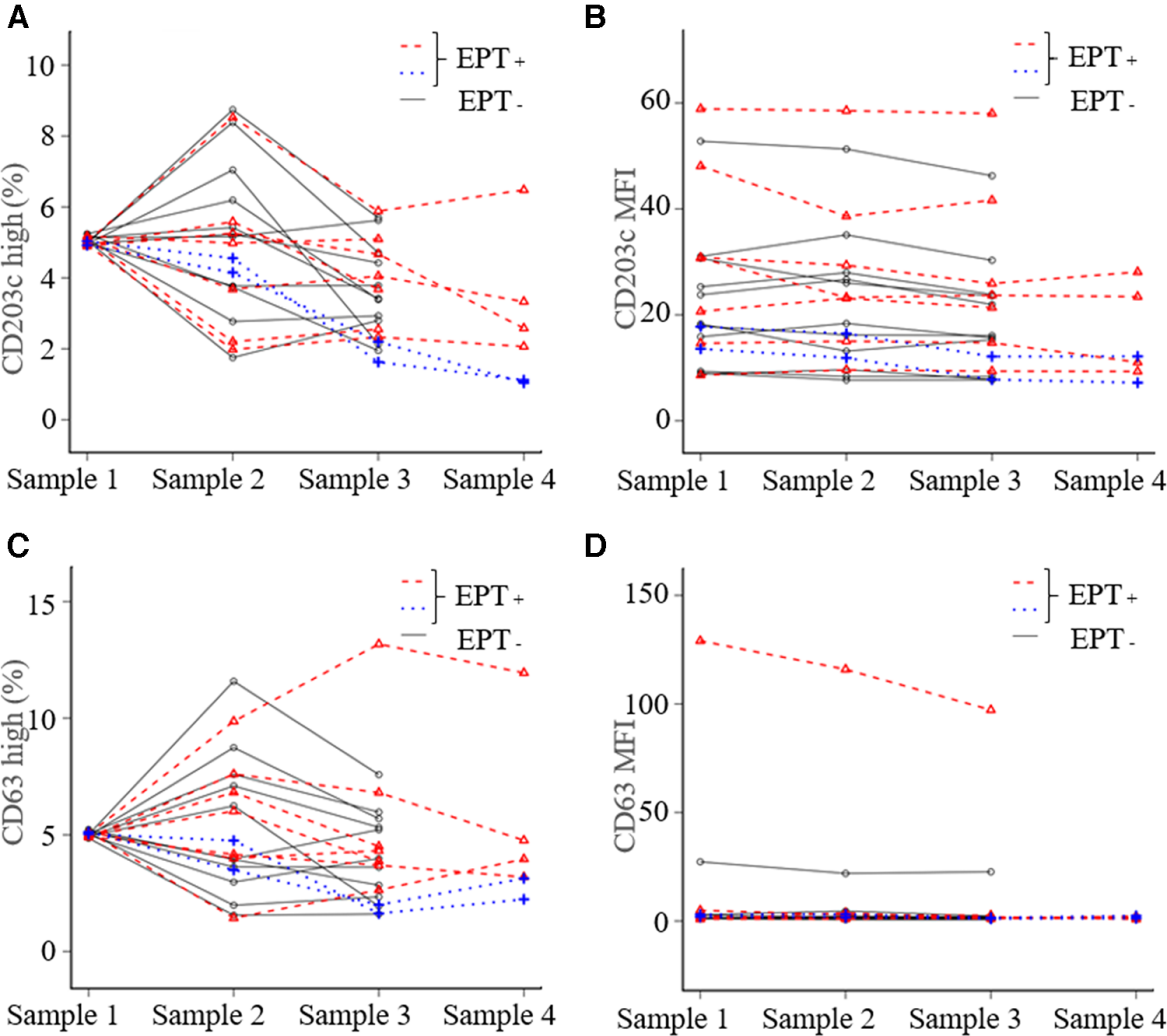
In vivo basophil activation status during the exercise provocation test. The basophil activation status was evaluated based on the expression of activation markers (CD203c and CD63). We compared the changes in the activation status by both mean fluorescence intensity (MFI) and CD203c-high or CD63-high basophils, which were gated over the highest 5% of the expression in comparison to sample 1. (**A**) CD203c high, (**B**) CD203c MFI, (**C**) CD63 high, and (**D**) CD63 MFI. The blue dotted lines represent the patients who showed the highest and the second-highest TS. Sample 1; before eating, Sample 2; 30 min later, Sample 3; immediately after exercise, Sample 4; at the peak of the provoked symptoms. EPT, exercise provocation test.

**Figure 3:**
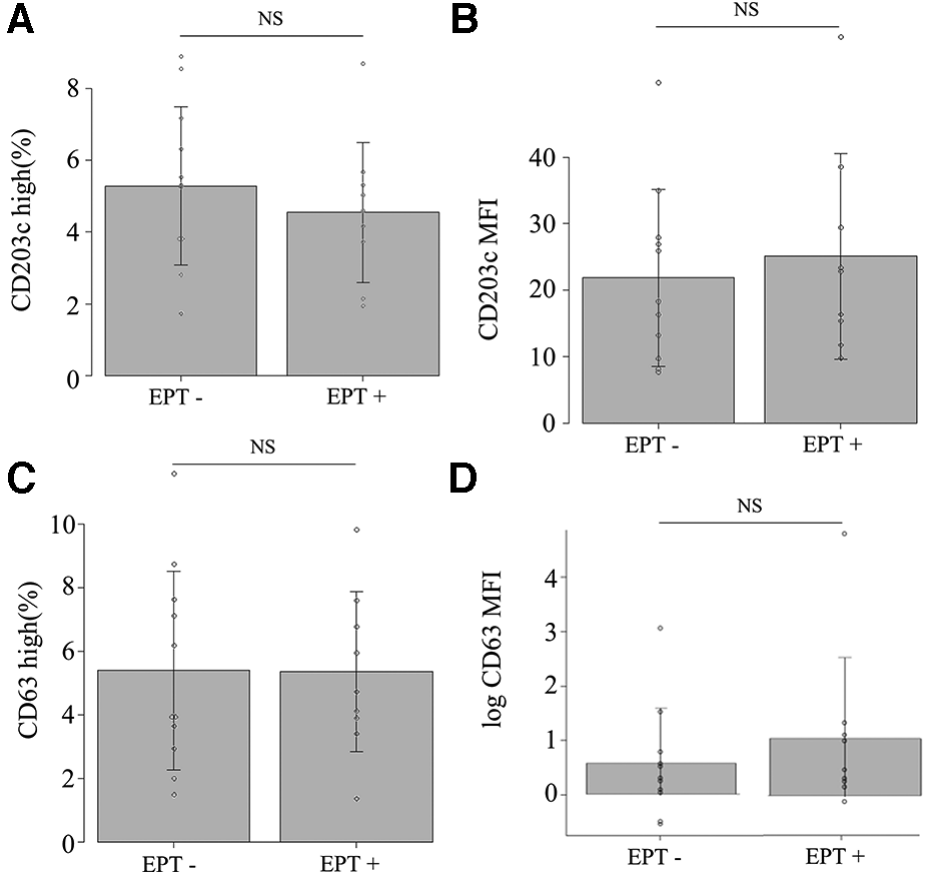
*In vivo* basophil activation at sample 2 (30 min after eating). (**A**) CD203c high, (**B**) CD203c MFI, (**C**) CD63 high, and (**D**) CD63 MFI. NS: not significant on the Mann–Whitney *U* test. EPT, exercise provocation test.

Nine (45%) of the 20 patients experienced provoked symptoms; the median TS was 10 points (5–40). None of the patients developed anaphylactic shock or needed an adrenaline injection. Basophil activation with *in vitro* stimulation in all patients under anti-IgE antibody and in 19 patients under the causative antigens was observed (data not shown). The expression of CD203c or CD63 and the MFI did not increase in samples 2, 3 or 4 compared to sample 1 (Figure 2). There was no significant difference between EPT-positive and negative subjects for all experimental outcomes (Figure 3 and Supplementary Figure S2). Of the nine EPT-positive patients, two patients—with the highest and second-highest TS—had levels over the standard in-plasma histamine levels: 27.0 and 8.8 ng/ml in samples 4 and 3, respectively. (The reference range is <1.24 ng/ml). However, these subjects did not demonstrate abnormally elevated tryptase levels (normal range <11.4 µg/L, Supplementary Figure 2).

## Discussion

Recent studies have reported that anaphylaxis is associated with reduced basophils in peripheral blood because activated basophils may migrate out of the peripheral blood (12). Other studies reported that basophil activation and degranulation might not occur in the peripheral blood, but in migrated tissues during anaphylaxis (8). These results indicate that most basophils migrate to the tissue that develops an allergic reaction. In our study, the patient with the highest TS had a gradually decreasing basophil count and did not have an increased expression of CD203c and CD63; this supports these previous described research findings (Figures 1, 4).

The association between the degranulation of basophils and the upregulation of CD203c and CD63 remains unclear. Increased expression of CD63 after ingestion of a red meat was shown not only in 9 out of 12 patients with α-gal allergy but also in 5 out of 13 healthy patients (7). Moreover, CD203c and CD63 expression of peripheral basophils was not upregulated, despite the histamine release (8, 13). Another study also demonstrated that patients with anaphylaxis showed no basophil activation in the peripheral blood, despite decreased intracellular histamine levels (12). In our study, the upregulation of CD203c and CD63 on basophils was not observed in EIARD-positive patients; these suggest that *in vivo* basophil activation after ingestion of the causative food may not be associated with EIARDs.

Consequently, detecting the activated basophils by CD203c or CD63 expression may not be a suitable method for predicting EIARDs and the exact cause of EIARDs remains unknown. Rather, we should consider determining basophil counts and factors involved in basophil migration, such as CCL2 (8) or factors causing severe allergic symptoms, such as PAF (13). Although no-stimulation basophil activation for patients with food allergies has already been reported, no consensus has been reached as to how this approach should be used. Keet et al. showed that MFI of the CD203c and CD63 on basophils under a no-stimulation condition significantly decreased with sublingual and oral immunotherapy for milk allergy (14). This suggests that evaluating no-stimulation basophil activation may be valid and effective in predicting whether or not the patients would be desensitized.

This study was exploratory and preliminary in nature; consequently, the sample size was small. Only a few patients developed severe allergic reactions, and experienced increases in total tryptase and plasma histamine, however, we could evaluate activation status of peripheral basophils during severe reaction of EIARDs in the patients.

However, to the best of our knowledge, this is the first study to evaluate basophil activation during EPT for patients with EIARDs. We failed to prove that peripheral basophils activate before—and even during—symptom provocation. We also did not evaluate the utility of basophil-activation status for diagnosing EIARDs; however, our findings may contribute to our understanding of the role of basophils in EIARDs.

## Data Availability

The raw data supporting the conclusions of this article will be made available by the authors, without undue reservation.
