# Supplementary figures and images for: Evaluation of peripheral basophil activation during exercise provocation test for desensitized patients
Source: Front Allergy. 2023 Dec 22;4:1298137. doi: 10.3389/falgy.2023.1298137 (PMC10770843; doi:10.3389/falgy.2023.1298137)

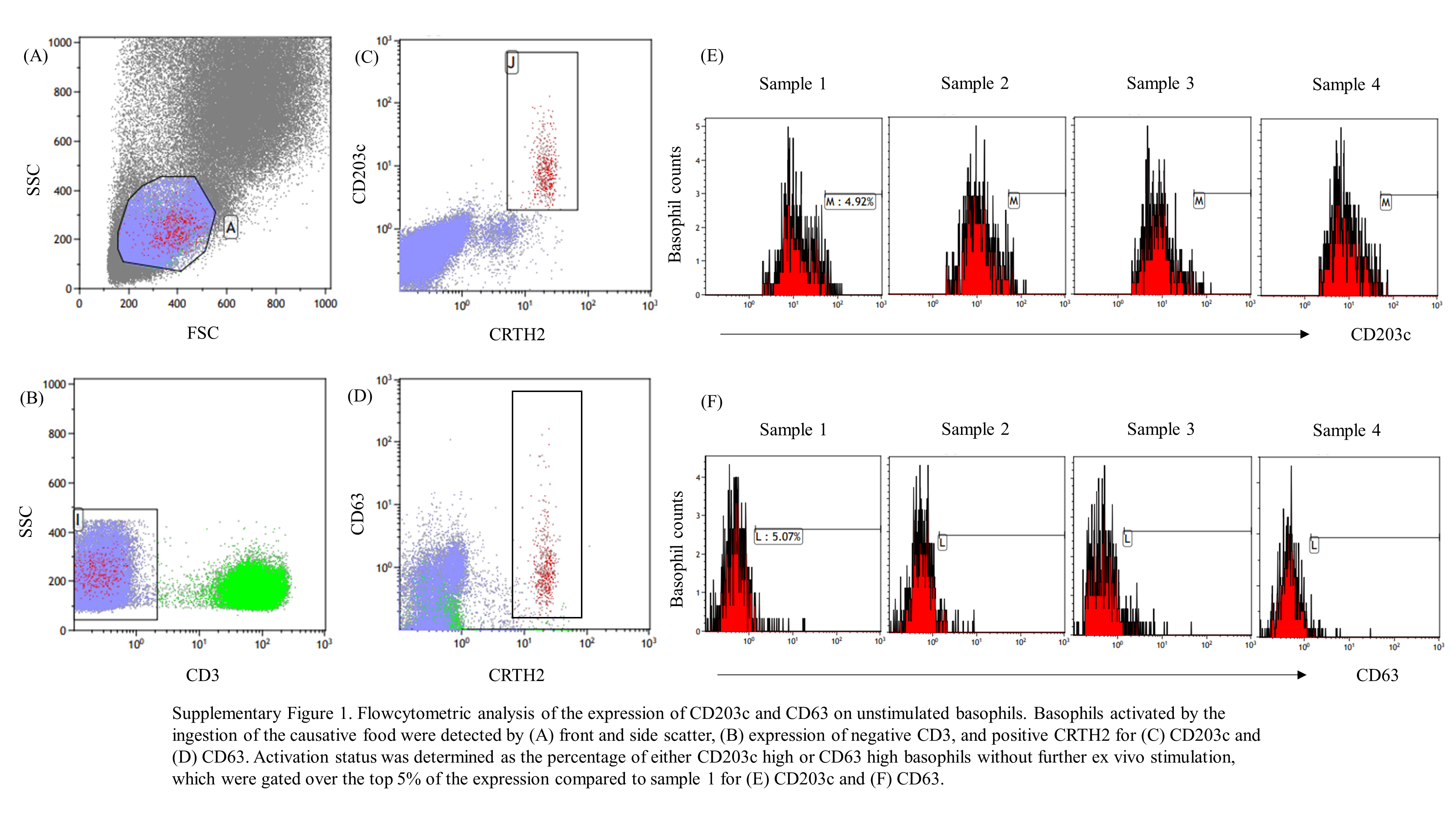

Supplement: Supplementary file 1 [file Image1.tif]

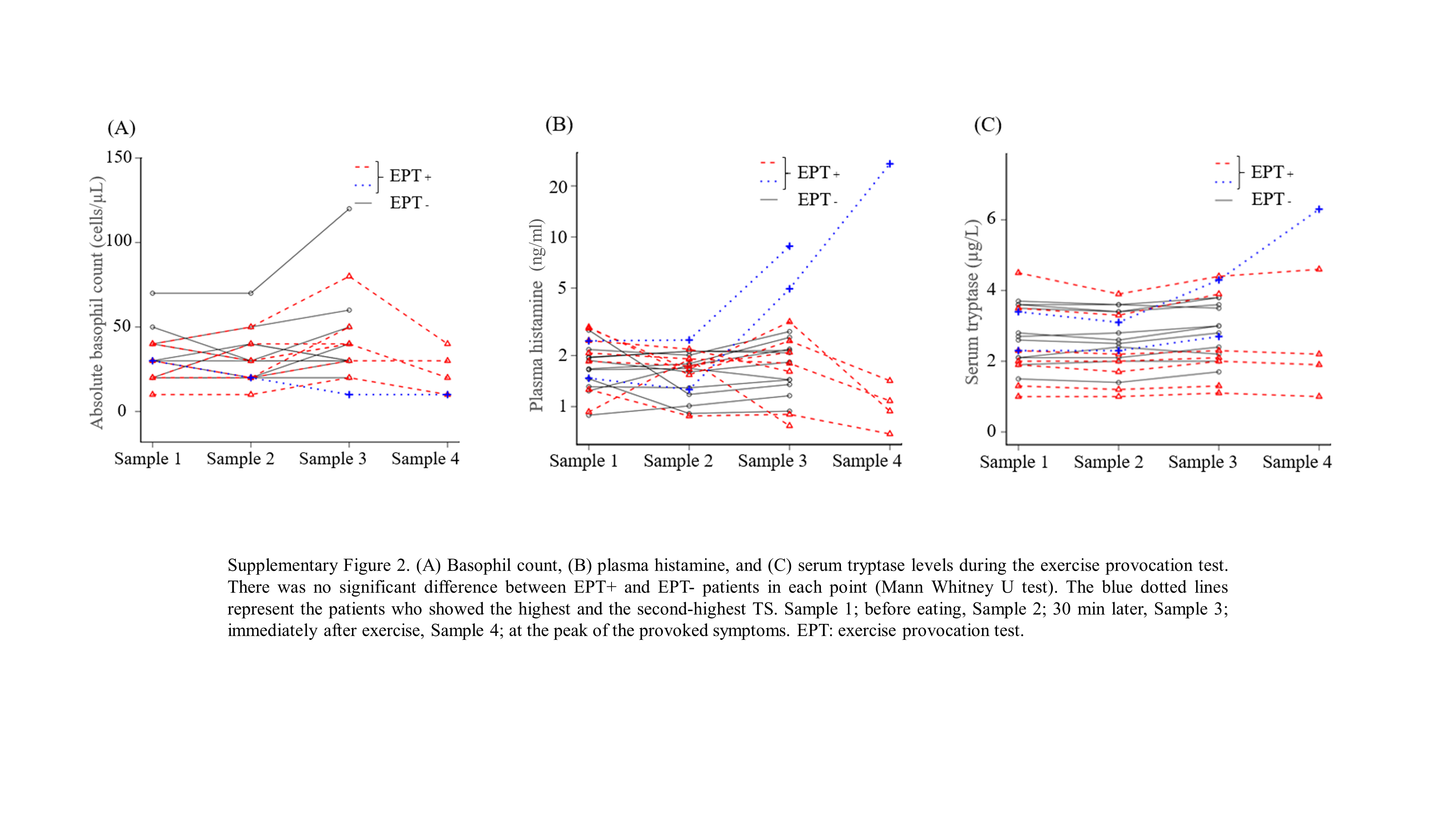

Supplement: Supplementary file 2 [file Image2.tif]
